# Phenotypic Characterization and Preliminary In Vivo Evaluation of a Locally Isolated *Klebsiella pneumoniae* Bacteriophage

**DOI:** 10.3390/biotech15040084

**Published:** 2026-09-29

**Authors:** Makhpal Sarmykova, Akbope Abdykalyk, Nazym Syrym, Bolat Yespembetov, Aktoty Anarbekova, Sabira Alpysbayeva, Azamat Abdimukhtar, Alinur Toleukhan, Kali Tileukhanov, Yeldos Serikbay, Bekzat Yerzhigit, Nazym Akimzhan, Kuandyk Zhugunissov, Sergazy Nurabayev

**Affiliations:** 1 Research Institute for Biological Safety Problems, National Holding QazBioPharm, LLP, Gvardeisky 080409, Kazakhstan; m.sarmykova@biosafety.kz (M.S.);; 2 Faculty of Veterinary Medicine and Zooengineering, Kazakh National Agrarian Research University, Abay Avenue 8, Almaty 050010, Kazakhstan

**Keywords:** *Klebsiella pneumoniae*, bacteriophage, phenotypic characterization, phage preparation, endotoxin reduction, antimicrobial resistance, guinea pig infection model

## Abstract

Drug-resistant *Klebsiella pneumoniae* is a serious clinical problem, and bacteriophages are being investigated again as antibacterial agents. This study describes the phenotypic characterization and a preliminary in vivo evaluation of vB_Kpn_NIIPB-V0006, a bacteriophage isolated locally on a drug-resistant clinical *K. pneumoniae* strain. The genome of the phage was not sequenced. It has therefore not been screened for lysogeny-associated, toxin, virulence or antimicrobial resistance genes, and the results below describe what the phage does, not whether it is suitable for therapy. We assessed plaque morphology, specific lytic activity, titer, host range on a limited non-target panel, particle morphology, adsorption, one-step growth, chloroform exposure, the frequency of phage-resistant host variants, endotoxin reduction, short-term safety in mice and antibacterial effect in guinea pigs. The phage lysed the target strain, with an Appelman activity of 10^−8^ and a titer of 1.7 × 10^11^ PFU/mL. Processing with 1-octanol lowered endotoxin content from 1200 to 12 EU/mL and preserved 80% of infective activity. Mice given the preparation intraperitoneally showed no mortality and no visible acute toxicity over 14 days. In guinea pigs with systemic *K. pneumoniae* infection, survival was the primary outcome: 0.5 mL three times daily gave 6/6 survivors against 1/6 in untreated controls (*p* = 0.015). Splenic bacterial burden, a secondary outcome, was also lower in that group (2.64 against 4.41 log_10_ CFU/g). Two features of the design bias this second comparison in favor of treatment, since survivors and non-survivors were sampled at different times, and phage being carried over into the plating step is likely to have lowered colony counts. The 1.77 log_10_ difference should therefore be read as an upper bound. The phage merits further study, but whole-genome sequencing, confirmation of the host species, host-range testing against diverse clinical isolates and a controlled comparison with antibiotic treatment are all needed before any therapeutic claim can be made.

## 1. Introduction

Drug-resistant *Klebsiella pneumoniae* remains a major clinical problem, particularly when extended-spectrum beta-lactamase-producing or carbapenem-resistant strains are involved. *K. pneumoniae* is an opportunistic member of the *Enterobacterales* and is associated with a range of nosocomial and invasive infections [1,2,3,4]. Its clinical importance reflects capsule-mediated virulence, biofilm formation, and the acquisition of resistance genes through mobile genetic elements [3,4,5,6].

Antimicrobial resistance surveillance remains limited in Central Asia, with reported gaps in stewardship and detection of resistant bacterial infections in Kazakhstan [7,8]. This has a direct consequence for the development of alternative antibacterial approaches. Because bacteriophages are strain-specific, a phage preparation is useful only where it matches the bacterial populations actually circulating in the region where it will be applied; phage collections assembled elsewhere cannot be assumed to cover locally prevalent strains. Where surveillance data are sparse, that match cannot be predicted from published information and must be established empirically, by isolating phages on locally obtained clinical strains and characterizing them directly. This is the rationale for the present work.

Bacteriophages are being reconsidered as antibacterial agents against multidrug-resistant bacteria because they can infect and lyse specific bacterial hosts and are not directly affected by classical antibiotic resistance mechanisms [9,10]. Interest extends beyond planktonic infection to biofilm-associated bacteria, where phages and phage-encoded depolymerases can degrade the extracellular polymeric matrix that limits antibiotic penetration [11]. However, their narrow host range requires careful characterization of each candidate phage. Key early parameters include plaque morphology, lytic activity, titer, adsorption, one-step growth kinetics, host range, stability, and emergence of phage-resistant variants [9,10,12].

For phages propagated on Gram-negative bacteria, preparation quality is also important because residual lipopolysaccharide can affect safety observations and animal experiments [13,14,15]. Whole-genome sequencing is increasingly required to assess the absence of lysogeny-associated genes, toxin genes, virulence-associated genes, and antimicrobial resistance genes [13,16,17]. Studies without phage genome data, including the present one, must therefore be interpreted as preliminary phenotypic evaluations and not as assessments of therapeutic suitability.

The aim of this study was to characterize, phenotypically, a locally isolated bacteriophage active against a drug-resistant clinical *K. pneumoniae* strain (plaque morphology, lytic activity, titer, host range, particle morphology, adsorption and one-step growth kinetics, chloroform sensitivity and the frequency of phage-resistant host variants) and to obtain preliminary in vivo data on endotoxin reduction after processing, short-term safety in mice and antibacterial effect in a guinea pig model of systemic infection.

## 2. Materials and Methods

### 2.1. Study Design

The study evaluated phage characteristics, endotoxin reduction as a preparation-quality step, short-term safety in mice, and preliminary antibacterial efficacy in guinea pigs. Phage administration was initiated 2 h after bacterial challenge and continued for 5 days. Survival was the primary outcome; splenic bacterial burden was a secondary outcome.

### 2.2. Animal Ethics and Welfare

Animal procedures were approved by the Local Bioethics Committee of the Research Institute for Biological Safety Problems on 4 July 2023 (Protocol No. 1) and were conducted in accordance with institutional requirements for the care and use of laboratory animals.

### 2.3. Bacterial Strain and Identification

A clinical *Klebsiella pneumoniae* strain, designated KP-MB-KAZ34, was used as the host strain for bacteriophage propagation and as the challenge strain in the animal infection model. The strain was obtained from de-identified clinical material and stored at −80 °C in glycerol (neoFroxx GmbH, Einhausen, Germany) and GRM broth at a 1:1 ratio.

Before use, the strain was revived on GRM agar and cultured in GRM broth at 37 °C for 18–24 h. GRM broth is a dry nutrient medium based on fish-meal hydrolysate (State Research Center for Applied Microbiology and Biotechnology, Obolensk, Russia), containing pancreatic hydrolysate of fish meal 24.0 g/L and sodium chloride 4.0 g/L; GRM agar has the same composition with the addition of 10.0 g/L microbiological agar. It is a general-purpose nutrient medium functionally equivalent to Luria–Bertani (LB) or nutrient broth for the propagation of Enterobacterales, and laboratories without access to GRM may substitute LB broth and LB agar. Antimicrobial susceptibility testing was performed exclusively on Mueller–Hinton agar, in accordance with EUCAST requirements. Primary identification was based on colony morphology, Gram staining, and catalase and oxidase tests.

Genomic DNA was extracted from an overnight culture of KP-MB-KAZ34 using the innuPREP DNA Mini Kit (Analytik Jena AG, Jena, Germany) according to the manufacturer’s instructions. The 16S rRNA gene was amplified using the universal bacterial primers 27F (5′-AGAGTTTGATCMTGGCTCAG-3′) and 1492R (5′-TACGGYTACCTTGTTACGACTT-3′). Amplicons were sequenced in both directions by the Sanger dideoxy chain-termination method using the BigDye Terminator v3.1 Cycle Sequencing Kit (Applied Biosystems, Austin, TX, USA); the primers used for amplification were also used for sequencing. The sequencing reaction products were purified using the BigDye XTerminator Purification Kit (Applied Biosystems, Foster City, CA, USA) and analyzed on a 3130xl Genetic Analyzer (Hitachi, Tokyo, Japan). The resulting nucleotide sequence chromatograms were assembled, edited, and analyzed using Sequencher version 5.4 (Gene Codes Corporation, Ann Arbor, MI, USA). The consensus sequence was compared with the NCBI GenBank nucleotide database using BLASTN v2.16.0 (National Center for Biotechnology Information, Bethesda, MD, USA) and was deposited under accession number PZ656399. No phylogenetic analysis was performed, because 16S rRNA gene sequences do not resolve the species of the *K. pneumoniae* complex (Section 4.5).

The limitations of this identification are acknowledged explicitly. The 16S rRNA gene does not reliably discriminate *K. pneumoniae* from the closely related species *K. quasipneumoniae* and *K. variicola*, and no MALDI-TOF mass spectrometry, *rpoB* sequencing or whole-genome sequencing was performed. The designation of KP-MB-KAZ34 as *K. pneumoniae* is therefore provisional; strictly, the isolate is a member of the *K. pneumoniae* species complex, and definitive assignment requires one of the methods named above. This limitation applies to every statement about the host strain in this article.

### 2.4. Antimicrobial Susceptibility Testing

Antimicrobial susceptibility testing of *K. pneumoniae* KP-MB-KAZ34 was performed by the disk diffusion method on Mueller–Hinton agar (TM Media, Titan Biotech Ltd., Bhiwadi, India) according to EUCAST version 15.0 [18]. Antimicrobial disks (HiMedia Laboratories Pvt. Ltd., Mumbai, India) contained ampicillin 10 µg, amoxicillin–clavulanic acid 20/10 µg, cefotaxime 5 µg, ceftazidime 10 µg, cefepime 30 µg, imipenem 10 µg, meropenem 10 µg, ciprofloxacin 5 µg, levofloxacin 5 µg, gentamicin 10 µg, and amikacin 30 µg.Inhibition-zone diameters were interpreted using EUCAST breakpoints for Enterobacterales. *Escherichia coli* ATCC 25922 (American Type Culture Collection, Manassas, VA, USA) was used for quality control.

### 2.5. Bacteriophage Isolation and Preparation

The bacteriophage evaluated in this study is designated vB_Kpn_NIIPB-V0006 (deposited in the institutional collection as *Klebsiella pneumoniae* bacteriophage NIIPB/V-0006). The designation follows the structured nomenclature convention for bacteriophages; no morphotype letter is included, in line with the abolition of morphology-based taxa by the ICTV [17], and no taxonomic assignment is implied in the absence of genome data. It was isolated from environmental material, including soil samples, by enrichment on *K. pneumoniae* KP-MB-KAZ34. Briefly, approximately 5 g of environmental material was suspended in 45 mL of GRM broth, supplemented with 5 mL of an actively growing *K. pneumoniae* host culture, and incubated at 37 °C for 24 h with shaking at approximately 120 rpm. After enrichment, the suspension was centrifuged at 4000 rpm for 10 min, treated with chloroform to a final concentration of 0.1%, and filtered sequentially through 0.45 µm and 0.22 µm membrane filters. The sterile filtrate was used for phage propagation and subsequent characterization.

For preparation of the working phage stock, the phage was propagated on the indicator *K. pneumoniae* strain. After visible lysis, the lysate was clarified by centrifugation, filtered through a 0.22 µm membrane filter, and stored at 2–8 °C until use.

### 2.6. Phage Activity, Titer and Plaque Morphology

Phage activity was first screened by spot test on a soft-agar lawn of an 18–24 h *K. pneumoniae* culture at 37 °C for 18–24 h; clear lysis zones were scored as positive. Specific lytic activity was determined by the Appelman method, in which serial ten-fold dilutions of the phage suspension are added to a broth culture of the host strain and the highest dilution still producing visible lysis is recorded; the result is therefore expressed as a dilution and not as a concentration. Titer was determined by the Gratia double-layer agar method [19]: 0.1 mL of each dilution was mixed with host culture and soft agar and incubated at 37 °C for 18–24 h. Based on 170 plaques at the 10^−8^ dilution, the working titer was 1.7 × 10^11^ PFU/mL. Plaque morphology was recorded after incubation, including inspection for translucent halos surrounding plaques, which are indicative of capsule-degrading depolymerase activity.

### 2.7. Host Range Testing

Lytic activity was tested against the host *K. pneumoniae* strain and against single isolates of three non-target species: *Escherichia coli, Pseudomonas aeruginosa* and *Enterococcus faecium*. Phage suspension was spotted onto soft-agar overlay lawns of each strain; visible lysis after 18–24 h at 37 °C was scored as positive. This panel comprises one isolate per non-target species and contains no *K. pneumoniae* isolates other than the propagating host. It is therefore not sufficient to establish host specificity or to define a lytic spectrum [12,20] and the results are reported only as the outcome of testing under these limited conditions.

### 2.8. Transmission Electron Microscopy

Phage morphology was examined by transmission electron microscopy using a JEM-100 CX-II microscope (JEOL, Tokyo, Japan) at an accelerating voltage of 80 kV and a magnification of 20,000×. Phage suspension was applied to carbon-coated copper grids and negatively stained according to the laboratory protocol. Head and tail dimensions were measured from calibrated micrographs using the web-based version of ImageJ v1.54 (National Institutes of Health, Bethesda, MD, USA) and are reported as mean ± SD of four particles. In the absence of whole-genome sequencing, the phage is described only as a tailed bacteriophage, and no morphotype-based taxonomic assignment is made (Section 3.3).

### 2.9. Chloroform Exposure and Resistant-Variant Frequency

Sensitivity to chloroform (EKOS-1, Staraya Kupavna, Russia) was evaluated by exposing the phage suspension to chloroform at a final concentration of 10% (*v*/*v*) for 15, 30, 45 and 60 min at room temperature. Following each exposure period, the samples were centrifuged at approximately 7000× *g* for 15 min at 4 °C to achieve phase separation. The upper aqueous phase containing the phage particles was carefully collected without disturbing the chloroform phase and was immediately subjected to serial dilution and titration by the double-layer agar method. Residual phage activity was assessed from plaque formation on the lawn of the host *K. pneumoniae* strain. A confluent bacterial lawn was observed on all assay plates, including those on which no plaques were detected, indicating that the absence of plaques reflected loss of detectable phage infectivity rather than inhibition of bacterial growth by residual chloroform.

The frequency of emergence of phage-resistant bacterial variants was determined by exposing the host *K. pneumoniae* population to the phage at a multiplicity of infection (MOI) of 1. The bacterial suspension and phage preparation were mixed, incorporated into soft agar, and overlaid onto solid agar plates, which were incubated at 37 °C for 24 h. Colonies growing in the presence of the phage were counted as putative phage-resistant variants, and the frequency of resistance emergence was calculated as the number of colonies recovered under phage pressure divided by the total number of viable bacterial cells determined from the corresponding phage-free control. The recovered colonies originated from the initially characterized pure *K. pneumoniae* culture; however, no species-level identification of individual colonies was performed, and they are therefore referred to as putative phage-resistant variants rather than independently confirmed resistant variants.

### 2.10. Adsorption and One-Step Growth Analysis

Adsorption was assessed by measuring the proportion of free phage remaining in suspension over time. A fresh 18–24 h *K. pneumoniae* culture was mixed with the phage suspension at a multiplicity of infection (MOI) of 0.01 and incubated at 37 °C. At selected time points, cells were separated by centrifugation, and the free phage titer in the supernatant was determined by the double-layer agar method. Adsorption was expressed as the percentage of free phage remaining relative to the count at time zero.

For one-step growth analysis, the phage was mixed with a fresh host culture at an MOI of 0.01 and allowed to adsorb at 37 °C for approximately 10 min. Non-adsorbed phage was reduced by dilution and/or separation, and the suspension was incubated at 37 °C. Sampling was not continued beyond 120 min, so no data are available on bacterial regrowth or on phage performance during prolonged co-incubation.

### 2.11. Endotoxin Reduction and Quality Control

Because the phage was propagated on the Gram-negative host *K. pneumoniae*, endotoxin reduction was performed before animal administration. The crude phage lysate was clarified by centrifugation and filtered through a 0.22 µm membrane filter (Merck Millipore, Darmstadt, Germany). Endotoxin reduction was then performed by 1-octanol (Sigma-Aldrich, St. Louis, MO, USA) extraction, followed by buffer exchange and final sterile filtration through a 0.22 µm membrane filter. The resulting preparation was referred to as the endotoxin-reduced phage preparation. Residual 1-octanol was not quantified in the final preparation.

Endotoxin concentration and infective phage titer were measured before and after endotoxin reduction. Endotoxin concentration was determined using a chromogenic Limulus amebocyte lysate assay according to the manufacturer’s instructions. Endotoxin-free consumables and pyrogen-free water were used throughout the assay. Samples were tested in serial dilutions to reduce matrix interference. No further quality-control parameters (sterility testing beyond filtration, residual host-cell protein and DNA, formulation stability, batch-to-batch reproducibility) were assessed.

### 2.12. Short-Term Safety Assessment in Mice

Short-term safety was evaluated in 40 clinically healthy young adult female CD-1 (ICR) outbred mice weighing 18–22 g. Animals were divided into four groups of 10 animals each. Mice in the experimental groups received the endotoxin-reduced phage preparation intraperitoneally at dose volumes of 0.1, 0.3, or 0.5 mL, corresponding to 1.36 × 10^10^, 4.08 × 10^10^ and 6.80 × 10^10^ PFU per mouse, respectively. Control mice received 0.5 mL of sterile physiological saline by the same route. Given the residual endotoxin concentration of 12 EU/mL in the endotoxin-reduced preparation, the corresponding endotoxin loads were 1.2, 3.6, and 6.0 EU per animal for the 0.1, 0.3, and 0.5 mL dose volumes, respectively. These correspond to approximately 60, 180 and 300 EU/kg for a 20 g mouse, and therefore exceed the threshold conventionally applied to parenteral products for human use (5 EU/kg per hour) by one to two orders of magnitude. This is stated here so that the safety observations reported in Section 3.6 are interpreted accordingly. No endotoxin-only control group (equivalent residual endotoxin without phage) and no vehicle-only control containing the residual solvent were included; the saline control therefore does not allow the contribution of residual endotoxin or 1-octanol to be separated from that of the phage. Animals were observed daily for 14 days for mortality and visible clinical signs of toxicity.

### 2.13. Guinea Pig Infection Model

Preliminary antibacterial efficacy was evaluated in guinea pigs infected with *K. pneumoniae* KP-MB-KAZ34. Seventy-eight clinically healthy young adult female guinea pigs weighing approximately 367–408 g at the start of the experiment were allocated to 13 groups of six animals using simple random allocation after acclimatization. Animals were housed under standard conditions with free access to food and water and acclimatized before the experiment; none were excluded after the start of the experiment. Humane endpoints were severe inactivity, inability to access food or water, marked respiratory distress, and loss of responsiveness; animals reaching them were euthanized with an intravenous overdose of sodium pentobarbital, and death was confirmed by absence of respiration, heartbeat, and corneal reflex.

A fresh 18–24 h culture was used to prepare a suspension at 1 × 10^7^ CFU/mL, and each animal was infected intraperitoneally with 0.2 mL, corresponding to 2 × 10^6^ CFU. Two hours after infection, treatment groups received the endotoxin-reduced phage preparation intraperitoneally for 5 consecutive days at 0.1, 0.2, 0.3, or 0.5 mL per administration, corresponding to 1.36 × 10^10^, 2.72 × 10^10^, 4.08 × 10^10^, and 6.80 × 10^10^ PFU, respectively. The preparation was administered once, twice, or three times daily.

The design included an infected untreated control group but no antibiotic-treated comparator, no phage-only (uninfected) group, and no endotoxin- or vehicle-only group. Since KP-MB-KAZ34 remained susceptible to amikacin, an amikacin-treated group would have provided a clinically meaningful reference for the observed effect; its absence is a limitation of the study and is discussed in Section 4. Animals in the three-times-daily groups received up to 15 intraperitoneal injections over 5 days, each delivering up to 6.0 EU of residual endotoxin. Outcome assessment was not blinded.

Animals were monitored daily for survival, body weight, activity, posture, coat condition, response to handling, respiratory signs, food and water intake, and signs of pain or distress.

### 2.14. Splenic Bacterial Burden

Spleens were collected from surviving animals on day 5 after infection and from animals that died or reached humane endpoints at the time of death or euthanasia. Each spleen was weighed and homogenized in sterile phosphate-buffered saline to a final volume of 1.0 mL. Ten-fold serial dilutions were prepared, and 0.1 mL aliquots were plated onto nutrient agar. Plates were incubated at 37 °C for 24–48 h, and colonies with morphology consistent with *K. pneumoniae* were counted.

Bacterial burden was calculated as CFU/g using the formula shown below, where *N* is the number of colonies, *D* is the dilution factor, and m is the spleen mass in grams. Results were log10-transformed and expressed as log10 CFU/g.

CFU/g = *N* × *D* × 10/m,

Two methodological features of this procedure limit its interpretation. First, spleen homogenates from phage-treated animals contain phage as well as bacteria, and no step was included to remove or neutralize phage before plating (for example anti-phage serum, ferrous ammonium sulfate treatment, washing of bacterial pellets, or dilution beyond the effective phage concentration). Continued phage activity in the agar would reduce colony counts in treated animals independently of the bacterial burden present in vivo, biasing counts downward in exactly the groups in which a treatment effect is being sought. Second, samples were obtained at two different times, day 5 in survivors and the time of death or euthanasia in non-survivors, so group means combine animals at different stages of infection. Both features are considered in the interpretation of the results (Section 4).

### 2.15. Statistical Analysis

Statistical analysis was performed using GraphPad Prism version 10 (GraphPad Software, Boston, MA, USA). Because of the small group size (*n* = 6 per group), non-parametric methods were used throughout, and no test of normality was applied.

Survival on day 5 was the primary outcome. It was analyzed as the proportion of animals alive on day 5 and compared using Fisher’s exact test. The comparison between the best-performing regimen and the infected untreated control group was defined a priori as the primary comparison; all other survival comparisons were exploratory and are reported without adjustment for multiplicity.

Splenic bacterial burden was treated as a secondary outcome. It was summarized as mean ± SD and compared between the best-performing regimen and the infected untreated control group using the Mann–Whitney U test; all remaining between-group differences are described descriptively. A sensitivity analysis restricted to animals sampled on day 5 was considered but could not be performed, because only one animal in the infected untreated control group survived to day 5. In the absence of such an analysis, and given the phage carry-over effect described in Section 2.14, between-group differences in splenic burden are interpreted as upper bounds on the true effect rather than as unbiased estimates.

Body-weight change was analyzed only in animals surviving to day 5, over the common interval from day 0 to day 5. Measurements taken at death or euthanasia in non-survivors span different and much shorter intervals and were not pooled with day-5 measurements; they are reported separately and descriptively.

A two-sided *p*-value of <0.05 was considered statistically significant.

## 3. Results

### 3.1. Identification and Antimicrobial Resistance Profile of the Clinical Klebsiella pneumoniae Strain

A clinical *Klebsiella pneumoniae* isolate, designated KP-MB-KAZ34, was used as the host strain for bacteriophage propagation and as the challenge strain in the animal infection model. The isolate formed mucoid colonies typical of *K. pneumoniae* and appeared as Gram-negative rods by microscopy. The strain was catalase-positive and oxidase-negative. Species-level assignment was based on partial 16S rRNA gene sequencing (708 bp). BLASTN analysis against the NCBI nucleotide database returned 100% identity and 100% query coverage to *Klebsiella pneumoniae* sequences, and the sequence was deposited in GenBank under accession number PZ656399. Because the 16S rRNA gene does not discriminate *K. pneumoniae* from *K. quasipneumoniae* and *K. variicola*, this assignment is provisional, as set out in Section 2.3.

Antimicrobial susceptibility testing showed that KP-MB-KAZ34 was resistant to ampicillin, amoxicillin-clavulanic acid, cefotaxime, ceftazidime, cefepime, imipenem, meropenem, ciprofloxacin, levofloxacin, and gentamicin, while remaining susceptible to amikacin. Resistance across multiple antimicrobial classes indicated a multidrug-resistant (MDR) phenotype. Resistance to both imipenem and meropenem places KP-MB-KAZ34 in the carbapenem-resistant *K. pneumoniae* (CRKP) category; the carbapenemase genes underlying this phenotype were not characterized in the present study. Full antimicrobial susceptibility results are provided in Supplementary Table S1.

### 3.2. Isolation, Lytic Activity, Titer, and Plaque Morphology of vB_Kpn_NIIPB-V0006

**Figure 1:**
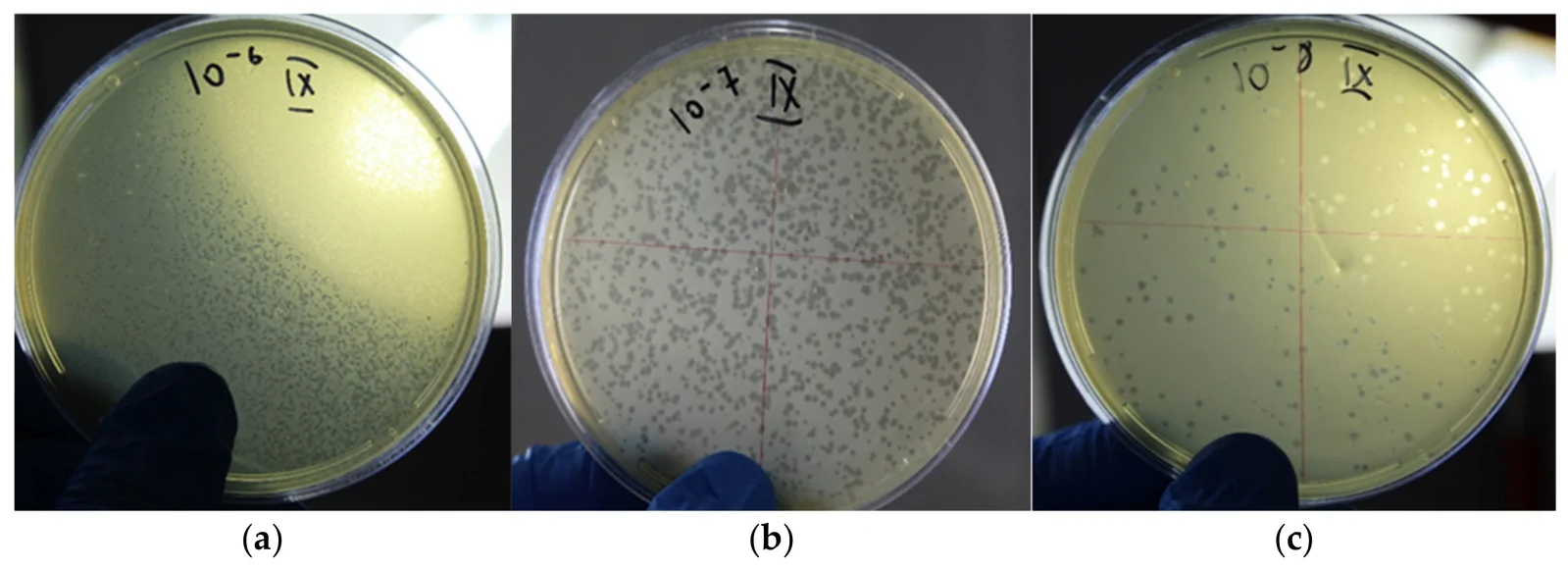
Plaque morphology and titration of vB_Kpn_NIIPB-V0006. (**a**) Dilution 10^−6^; (**b**) dilution 10^−7^; (**c**) dilution 10^−8^. Clear plaques were observed on the lawn of the indicator *Klebsiella pneumoniae* strain. The countable plate at the 10^−8^ dilution was used for titer calculation.

vB_Kpn_NIIPB-V0006 was isolated from environmental material by enrichment on *K. pneumoniae* KP-MB-KAZ34. The phage produced visible clear lysis zones in the spot test and formed clear plaques on the lawn of the indicator strain (Figure 1). Plaques were approximately 1.5–3.0 mm in diameter after 18–24 h of incubation at 37 °C. No translucent halos were observed around plaques under the conditions tested.

The Appelman activity was 10^−8^. In the double-layer agar assay, isolated plaques suitable for counting were observed at the 10^−8^ dilution, where 170 plaques were recorded. The calculated working titer was 1.7 × 10^11^ PFU/mL.

### 3.3. Host Range and Particle Morphology

No lytic activity was detected against the three non-target isolates tested (*Escherichia coli*, *Pseudomonas aeruginosa*, *Enterococcus faecium*); lysis was observed only against the propagating *K. pneumoniae* strain. Because the panel consisted of one isolate per non-target species and included no additional *K. pneumoniae* isolates, this result describes the outcome under the tested conditions only and does not establish host specificity or define the lytic spectrum of the phage.

**Figure 2:**
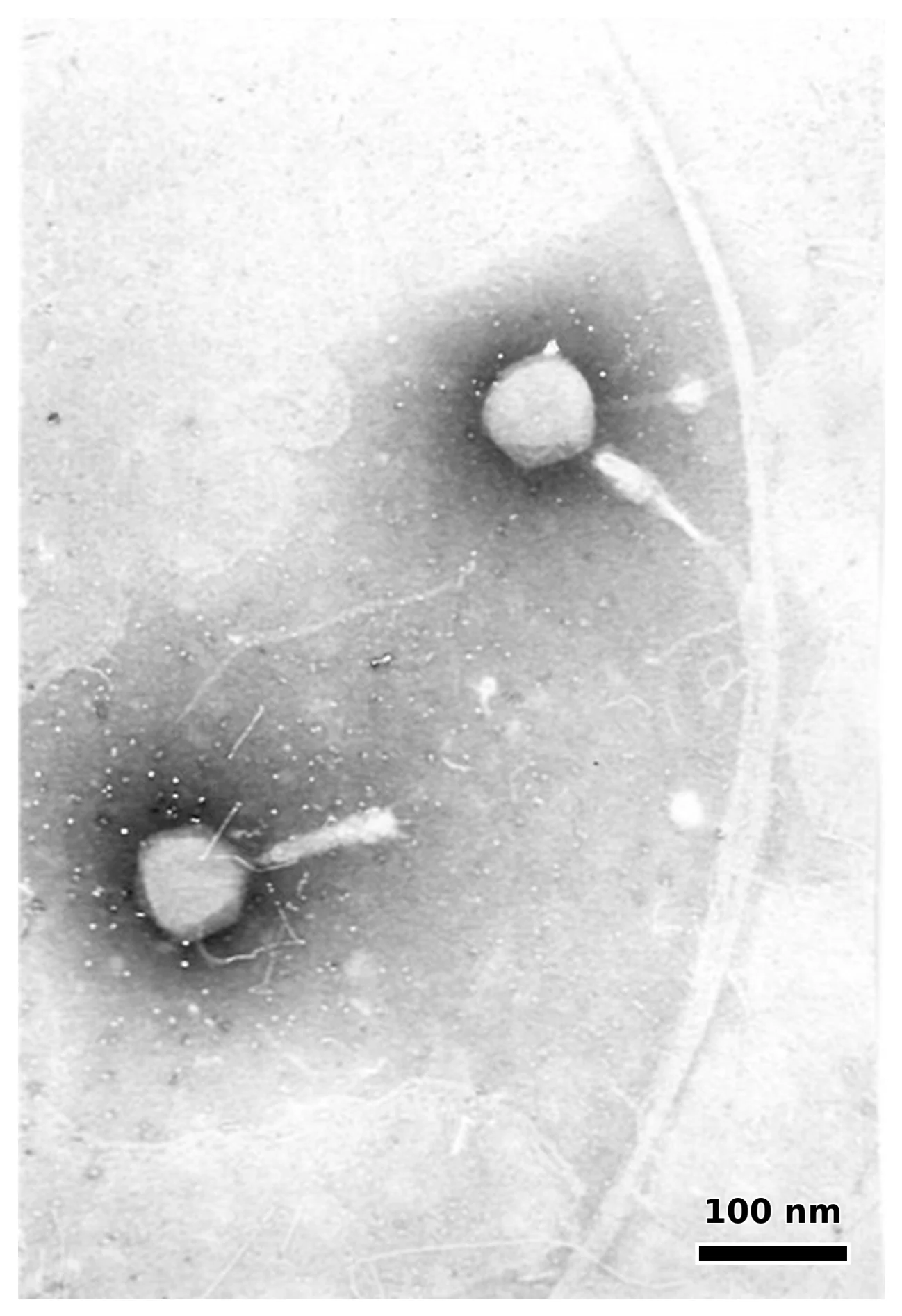
Transmission electron micrograph of vB_Kpn_NIIPB-V0006 after negative staining. Two representative particles of the four measured are shown, each with an isometric head and a tail; thin appendages consistent with tail fibers are visible at the distal end of the tail. Scale bar: 100 nm.

Transmission electron microscopy showed tailed bacteriophage particles with an isometric head and a tail (Figure 2). The head was hexagonal in outline, consistent with an icosahedral capsid, and measured 82 ± 5 nm between opposite vertices and 67 ± 3 nm between opposite faces. The tail measured 112 ± 13 nm in length and 18 ± 2 nm in width. Thin appendages consistent with tail fibers were visible at the distal end of the tail. In the absence of genome data, and following the abolition of morphology-based taxa [17], this description is provisional and no taxonomic assignment is made.

### 3.4. Adsorption, One-Step Growth Kinetics, Chloroform Exposure, and Resistant Variants

**Figure 3:**
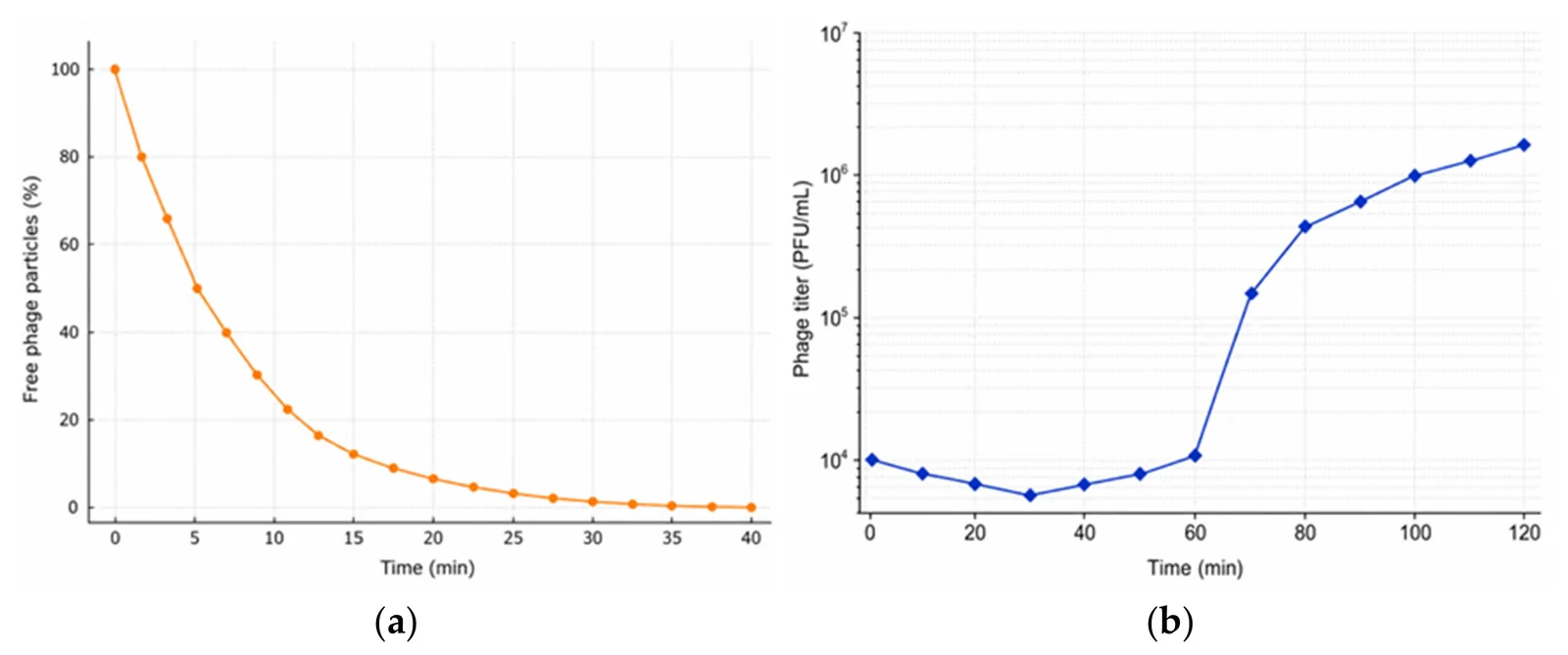
Adsorption and one-step growth kinetics of vB_Kpn_NIIPB-V0006. (**a**) Adsorption dynamics showing the decrease in free phage particles during incubation with the indicator *Klebsiella pneumoniae* strain. (**b**) One-step growth curve showing the increase in phage titer after the latent period.

Adsorption analysis showed rapid binding of the phage to the indicator *K. pneumoniae* strain (Figure 3a). Approximately 50% of free phage particles remained after 6–7 min, the free fraction fell below 10% by 20 min, and 2–3% of the initial free phage remained at 30 min.

One-step growth analysis showed no marked increase in extracellular phage titer during the first 60 min (Figure 3b). Phage release began at approximately 60–70 min, followed by an increase of about two orders of magnitude, from approximately 10^4^ to 1.7 × 10^6^ PFU/mL. The titer was still rising at 120 min, the end of the observation period, so no plateau was reached within the observation window. The corresponding burst size was approximately 170 PFU per infected cell, calculated as the ratio of the titer at 120 min to the titer during the latent period; because the curve had not plateaued, this is a lower-bound estimate.

The phage retained detectable activity after chloroform exposure for 15, 30, and 45 min, whereas no plaques were detected after 60 min. A confluent bacterial lawn was present on all assay plates, indicating that this reflected loss of phage infectivity rather than inhibition of the indicator strain by residual chloroform. The frequency of emergence of putative phage-resistant bacterial variants was 1.3 × 10^−5^.

### 3.5. Endotoxin Reduction and Preservation of Phage Infectivity

Endotoxin concentration decreased from 1200 EU/mL in the crude phage lysate to 12 EU/mL after 1-octanol-based processing, a 100-fold reduction. When normalized to phage titer, endotoxin content decreased from 7.06 to 0.088 EU/10^9^ PFU. The phage titer decreased from 1.7 × 10^11^ to 1.36 × 10^11^ PFU/mL, corresponding to 80% preservation of infective activity (Table 1).

### 3.6. Short-Term Safety in Mice

Short-term safety testing in mice showed no mortality during the 14-day observation period. Mice receiving the endotoxin-reduced phage preparation at 0.1, 0.3, or 0.5 mL remained clinically healthy. No severe depression, impaired coordination, respiratory distress, convulsions, visible local reactions, or other obvious signs of acute toxicity were recorded in any treated group. Control mice receiving sterile physiological saline also remained clinically healthy.

As noted in Section 2.12, these observations were made in animals receiving 1.2–6.0 EU of residual endotoxin per dose, and no endotoxin-only control was included; the results therefore indicate the absence of gross acute toxicity of the preparation as a whole and do not isolate the contribution of the phage itself.

### 3.7. Preliminary Antibacterial Efficacy in the Guinea Pig Infection Model

**Figure 4:**
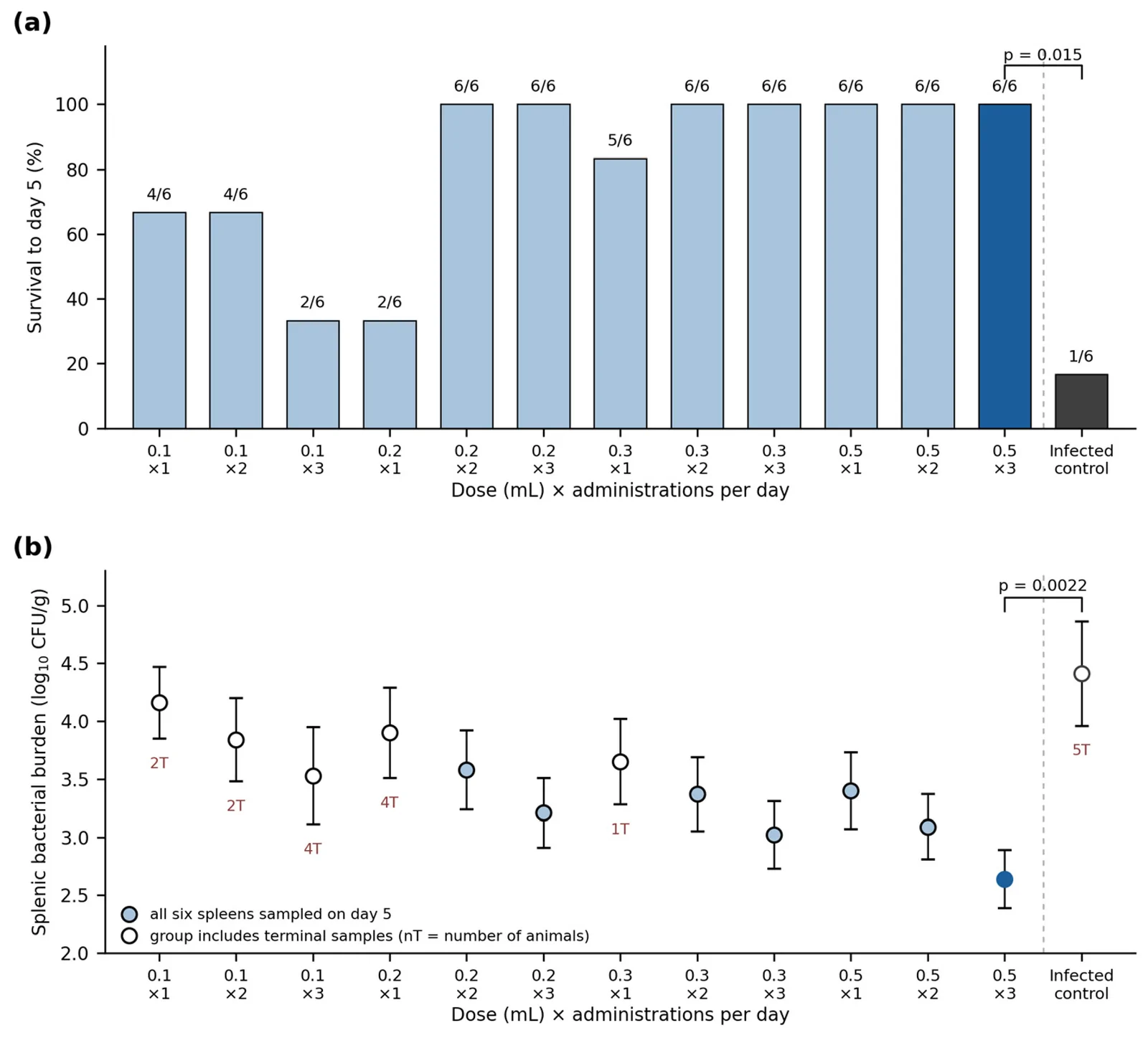
Preliminary antibacterial efficacy of vB_Kpn_NIIPB-V0006 in guinea pigs experimentally infected with *Klebsiella pneumoniae* (n = 6 per group). (**a**) Survival to day 5 in the infected untreated control group and in each phage-treated group, labeled by dose volume and number of administrations per day. The number of survivors is given above each bar. (**b**) Splenic bacterial burden, shown as group mean ± SD. Filled symbols indicate groups in which all six spleens were sampled on day 5; open symbols indicate groups that also include samples taken at death or at a humane endpoint, with the number of such animals given as nT. Because burden was measured at two different times, and because phage carried over into the plating step may lower colony counts in treated animals, between-group differences in panel (**b**) are interpreted as upper bounds rather than as unbiased estimates. *p* values refer to the primary comparison between the 0.5 mL three-times-daily group and the infected untreated control group (Fisher’s exact test for survival, Mann–Whitney U test for burden).

Untreated infected animals showed marked clinical deterioration, including reduced activity, body weight loss, poor coat condition, and respiratory abnormalities, and only 1/6 survived to day 5 (Figure 4).

Survival, the primary outcome, is shown in Table 2 and Figure 4a. Complete survival (6/6) was observed in seven groups (groups 5, 6, 8, 9, 10, 11 and 12), all of which received either one of the two highest dose volumes or twice- or three-times-daily administration at 0.2 mL.

The best outcome was observed in animals receiving 0.5 mL of the endotoxin-reduced phage preparation three times daily for 5 consecutive days, in which survival reached 6/6 animals and the mean splenic bacterial burden was 2.64 log_10_ CFU/g. In comparison, the infected untreated control group showed 1/6 survivors and a mean splenic bacterial burden of 4.41 log_10_ CFU/g.

Body weight was recorded daily. Body-weight change in animals surviving to day 5 and in animals that died or reached humane endpoints was not pooled, because the two groups were measured over different intervals: day 0 to day 5 in survivors, and day 0 to the last measurement before death or euthanasia in non-survivors. Animals that died or reached humane endpoints lost 13.5 ± 4.0% of their body weight over this shorter interval. Weight loss in animals surviving to day 5 was less pronounced, and was smallest in the 0.5 mL three-times-daily group; group means for survivors alone are not reported, because the number of contributing animals differs between groups and is as low as two in some groups.

The primary comparison between the 0.5 mL three-times-daily group and the infected untreated control group showed a significant difference in survival (6/6 vs. 1/6; Fisher’s exact test, *p* = 0.015). The corresponding splenic bacterial burden was also lower in the 0.5 mL three-times-daily group than in the infected untreated control group (2.64 ± 0.25 vs. 4.41 ± 0.45 log_10_ CFU/g; Mann–Whitney U test, *p* = 0.0022; Figure 4b), but this secondary outcome is affected by the two sources of bias described in Section 2.14 and Section 4 and should be read as an upper bound.

Survival did not increase monotonically with dose or frequency. At the lowest dose volume, three-times-daily administration (group 3) was associated with lower survival (2/6) than once- or twice-daily administration (4/6 each), and group 4 (0.2 mL once daily, 2/6) showed lower survival than group 1 (0.1 mL once daily, 4/6). Although complete survival was observed only in groups receiving higher doses or more frequent administration, the small group sizes precluded detection of a monotonic dose–response relationship (group 1 versus group 3, Fisher’s exact test, *p* = 0.57).

## 4. Discussion

This study provides a phenotypic characterization and preliminary in vivo evaluation of vB_Kpn_NIIPB-V0006, a locally isolated bacteriophage active against a drug-resistant clinical *Klebsiella pneumoniae* isolate. The phage showed clear lytic activity against the target strain, a high working titer, rapid adsorption, productive replication in one-step growth analysis, and preserved infective activity after endotoxin-reduction processing. In the animal experiments, the endotoxin-reduced preparation caused no mortality or visible acute toxicity in mice during 14 days of observation and improved survival in guinea pigs experimentally infected with *K. pneumoniae*.

The study is relevant because *K. pneumoniae* is a clinically important opportunistic pathogen with a strong capacity to acquire antimicrobial resistance determinants and cause difficult-to-treat infections [3,4,5,6]. KP-MB-KAZ34 showed resistance to several antimicrobial classes, including beta-lactams, carbapenems, fluoroquinolones, and gentamicin, while remaining susceptible to amikacin, which supports its use as a relevant target strain for preliminary evaluation of an alternative antibacterial approach [1,2,9,10].

### 4.1. Absence of Genome Data

The absence of whole-genome sequencing is the principal limitation of this work and constrains every other conclusion. Without genome data it is not possible to exclude lysogeny-associated genes, including integrase genes characteristic of temperate phages, nor toxin, virulence-associated or antimicrobial resistance genes. A phage carrying such genes would be unsuitable for therapeutic development regardless of how favorable its phenotypic profile appeared, and phenotypic evidence of clear-plaque formation does not exclude a temperate lifestyle. Current phage taxonomy and development rely on genome-based analysis, and genetic safety assessment is considered essential for phages intended for therapeutic use [13,16,17]. For this reason, the present findings are presented as a phenotypic evaluation, and no claim of therapeutic suitability is made. Whole-genome sequencing is the first required step in any further development of this isolate.

### 4.2. Preparation Quality and Residual Endotoxin

For phages propagated on Gram-negative bacteria, residual lipopolysaccharide is a critical confounder because it can influence animal safety observations and inflammatory outcomes [13,14,15]. In the present study, 1-octanol-based processing reduced endotoxin concentration 100-fold, from 1200 to 12 EU/mL, while preserving 80% of infective phage activity. Organic-solvent extraction is one of several available approaches; alternatives include polymyxin B affinity chromatography, anion-exchange and size-exclusion chromatography, ultrafiltration, caesium chloride density-gradient centrifugation, and Triton X-114 phase separation, which differ in achievable endotoxin clearance, phage recovery and scalability [14,15]. Chromatographic and affinity-based methods generally achieve lower residual endotoxin than single-step solvent extraction, and combining methods is common practice.

Whether 12 EU/mL is acceptable depends entirely on the intended use. For the intraperitoneal animal experiments reported here, loads of 1.2–6.0 EU per animal correspond to approximately 60–300 EU/kg in a 20 g mouse and approximately 3–15 EU/kg in a 400 g guinea pig. In the mouse experiments these values exceed the threshold conventionally applied to parenteral products for human use (5 EU/kg per hour) by more than one order of magnitude; in the guinea pig experiments they range from just below that threshold to three times above it. The preparation is therefore adequate for exploratory animal work but is not of clinical grade.

Residual endotoxin also bears on interpretation of the efficacy data. Animals in the three-times-daily groups received up to 15 intraperitoneal injections over 5 days, each containing residual endotoxin and trace organic solvent. Repeated intraperitoneal administration of such a preparation may cause cumulative peritoneal irritation and inflammatory responses which, superimposed on an established systemic infection, could plausibly worsen the outcome. This provides an alternative, non-random explanation for the reduced survival observed in group 3 (0.1 mL three times daily, 2/6) relative to the once- and twice-daily groups at the same dose volume (4/6 each), in which the phage dose was insufficient to offset the additional inflammatory burden. The present study cannot distinguish this explanation from chance variation, because no endotoxin-only or vehicle-only control was included and no inflammatory markers were measured. Future studies should include such control groups, measure cytokines, and quantify residual solvent in the final preparation.

### 4.3. Efficacy Readouts and the Magnitude of the Observed Effect

Survival, the primary outcome, showed a clear difference between the best-performing regimen and infected untreated controls (6/6 versus 1/6, *p* = 0.015). This readout is not affected by the sampling and plating problems discussed below and constitutes the most robust finding of the animal experiments. The most favorable regimen was 0.5 mL administered three times daily for 5 consecutive days. These results are consistent with previous experimental studies in which bacteriophage treatment improved outcomes in animal models of *K. pneumoniae pneumonia*, bacteremia, liver abscess, and systemic infection [21,22,23,24,25], and with broader evidence from Gram-negative ESKAPE infection models [26].

Splenic bacterial burden, the secondary outcome, requires more careful interpretation, because two independent features of the design bias it in the same direction, in favor of the treated groups. The first is sampling-time heterogeneity: burden was measured on day 5 in survivors and at death or euthanasia in non-survivors, so group means are not directly comparable. The imbalance is most severe in the primary comparison itself, where the control mean derives from one day-5 animal and five terminal animals, whereas the best-performing regimen contributed six day-5 animals. A sensitivity analysis restricted to day-5 samples would be the appropriate remedy but is impossible here, since the control group contributed only one such animal. The second is phage carry-over during plating: spleen homogenates from treated animals contain high concentrations of phage, which are transferred to the agar together with the bacteria and can continue to lyse them during incubation, lowering colony counts independently of the burden present in vivo. No neutralization or removal step was included, and this artifact operates only in treated groups.

Taken together, these two biases mean that the 1.77 log_10_ CFU/g difference between the best-performing regimen and the infected untreated control group should be regarded as an upper bound on the true effect rather than as an unbiased estimate. Consistent with this, the ranking of groups by burden does not follow the ranking by survival: group 3 showed a lower mean burden than group 1 despite lower survival. Survival and splenic burden are therefore best treated as partly independent readouts in this dataset rather than as a single consistent dose–response signal. Future work should use a single predefined sampling time common to all animals, include phage neutralization before plating, and analyze survival over the complete observation period. Pharmacokinetic analysis and delayed-treatment groups are also needed, since phage pharmacokinetics and pharmacodynamics are strongly influenced by bacterial burden, route of administration, timing of treatment, tissue distribution, and host clearance [27,28].

### 4.4. Capsule, Extracellular Polysaccharide and Durability of the Effect

KP-MB-KAZ34 formed distinctly mucoid colonies, and capsular polysaccharide and extracellular polymeric substances are well recognized barriers to phage adsorption in *K. pneumoniae*; phage efficacy may decline as this material accumulates, particularly in biofilm-associated infection [11]. The present study did not address this directly. No depolymerase assay, biofilm assay or capsular typing was performed, and lysis kinetics were not followed beyond 120 min, so no data are available on bacterial regrowth during prolonged co-incubation.

Two indirect observations are relevant. First, the phage adsorbed rapidly and formed clear plaques on lawns of this mucoid strain, indicating that capsule expression under the conditions tested did not prevent productive infection; however, no translucent halos were observed around plaques, so there is no phenotypic evidence of capsule-degrading depolymerase activity and the absence of halos does not exclude the presence of a depolymerase gene. Second, putative phage-resistant variants arose at a frequency of 1.3 × 10^−5^; capsule loss or alteration is among the most common routes to phage resistance in *K. pneumoniae*, but the recovered colonies were not characterized, so this frequency cannot be attributed to capsular change.

Determining whether the phage encodes a depolymerase requires the genome data identified in Section 4.1. Capsular typing of the host strain, biofilm assays against mature exopolysaccharide, and extended lysis kinetics with monitoring of bacterial regrowth are necessary before any claim about activity against mucoid or biofilm-associated *K. pneumoniae* can be made.

### 4.5. Host Identification, Host Range and Morphology

Species identification of the host strain rested on 16S rRNA gene sequencing alone, which does not resolve *K. pneumoniae* from *K. quasipneumoniae* or *K. variicola*. The designation used throughout this article is therefore provisional, and confirmation by MALDI-TOF mass spectrometry, *rpoB* sequencing or whole-genome sequencing is required. For a phage candidate this ambiguity is material, since host range is defined relative to the host species.

The host-range panel comprised one isolate each of three non-target species and no *K. pneumoniae* isolates beyond the propagating strain. This is not sufficient to characterize a lytic spectrum, and the absence of lysis observed here should not be read as evidence of specificity. Assessment of clinical potential requires testing against a substantial collection of *K. pneumoniae* clinical isolates of diverse origin and capsular type, together with a wider non-target panel; such testing will determine whether the phage is suitable only for narrow strain-specific application or for broader development, potentially within a cocktail [12,20].

Particle morphology was assessed by transmission electron microscopy and the phage was described as a tailed bacteriophage. Following the abolition of morphology-based taxa [17], morphology alone cannot support taxonomic classification, and the dimensions reported here are descriptive only.

### 4.6. Further Limitations

The study did not include an antibiotic-treated comparator. Because KP-MB-KAZ34 remained susceptible to amikacin, an amikacin-treated group was feasible and would have placed the observed phage effect in clinical context; without it, the efficacy reported here can be judged only against untreated infection, which is the least demanding possible comparison. This is a substantive limitation, and comparative studies including amikacin monotherapy and phage–antibiotic combination groups are needed.

The study also lacked delayed-treatment groups, cytokine or inflammatory-marker assessment, pharmacokinetic analysis, repeated-dose toxicology, and long-term safety evaluation. Outcome assessment was not blinded, and future studies should follow contemporary animal-reporting recommendations more closely, including blinding where feasible and predefined exclusion criteria [29]. Putative phage-resistant variants were detected, albeit at low frequency; their emergence should be characterized further, since resistance development may limit the effectiveness of monophage treatment. At the challenge inoculum used here (2 × 10^6^ CFU per animal), a frequency of 1.3 × 10^−5^ corresponds to approximately 26 pre-existing phage-insensitive cells at the time of infection. Resistant subpopulations were therefore present from the outset rather than arising only under treatment pressure, which is one reason why monophage preparations are generally considered less robust than cocktails. Characterization of the recovered variants, in particular whether resistance is capsule-associated and whether it carries a fitness cost, is required before further development.

Complete loss of infectivity after 60 min of exposure to 10% chloroform is unusual for a tailed bacteriophage, since tailed phages lack a lipid envelope and are generally chloroform-tolerant. Growth of the bacterial lawn on the assay plates excludes inhibition of the indicator strain as an explanation, so the observation appears to reflect a property of this phage, possibly related to lipid-containing structural components or to low structural stability; distinguishing between these would require structural and genomic analysis. Practically, chloroform should not be used in the preparation or storage of this phage, and the 0.1% chloroform step used during isolation should be reconsidered in future work.

Overall, this study supports further phenotypic and genetic investigation of vB_Kpn_NIIPB-V0006, and not its use as a therapeutic candidate. The work combines local phage isolation, biological characterization, endotoxin-reduction processing, short-term safety assessment and preliminary in vivo antibacterial evaluation. The next development steps should include whole-genome sequencing, definitive host-species identification, expanded host-range testing, characterization of phage-resistant variants, improved purification with defined release specifications, formulation stability testing, pharmacokinetic and toxicological assessment, and direct comparison with amikacin and phage–antibiotic regimens. These steps are necessary before therapeutic application can be considered.

## 5. Conclusions

vB_Kpn_NIIPB-V0006, a bacteriophage isolated locally on a drug-resistant clinical *Klebsiella pneumoniae* isolate, has been characterized phenotypically. It lyses its propagating host, adsorbs rapidly, replicates productively under one-step growth conditions, and retains 80% of its infective activity after 1-octanol-based endotoxin reduction that lowers endotoxin content 100-fold. The resulting preparation caused no observable acute toxicity in mice over 14 days and, in guinea pigs with established systemic infection, raised day-5 survival from 1/6 in untreated controls to 6/6 at the highest dose and frequency tested.

What this study does not establish is equally important. The genome has not been sequenced, so the phage cannot be excluded from carrying lysogeny-associated, toxin, virulence or resistance genes. The host species has not been confirmed beyond the *K. pneumoniae* species complex. The lytic spectrum has not been defined. The preparation is not of clinical grade. The reduction in splenic bacterial burden is an upper bound rather than an unbiased estimate of effect. The isolate is accordingly presented as a characterized local phage that merits further investigation, and not as a therapeutic candidate. The sequence of work required to change that assessment is defined and ordered: whole-genome sequencing first, because it gates the safety question and every downstream decision; then definitive identification of the host species; then host-range testing against a diverse collection of clinical isolates; and only then comparative in vivo studies against amikacin, using a preparation manufactured to defined release specifications.

## Figures and Tables

**Table 1:** Endotoxin content and phage titer before and after purification.

Sample	Phage Titer, PFU/mL	Endotoxin, EU/mL	Endotoxin, EU/10^9^ PFU	Phage Titer Preservation, %
Crude phage lysate	1.7 × 10^11^	1200	7.06	100
Endotoxin-reduced phage preparation	1.36 × 10^11^	12	0.088	80

**Table 2:** Survival and splenic bacterial burden in guinea pigs experimentally infected with *Klebsiella pneumoniae* and treated with the endotoxin-reduced phage preparation ^1^.

Group	Dose (mL)	Freq./Day	PFU/Administration	Splenic Burden, Mean ± SD (log_10_ CFU/g)	Sampled Day 5 (n)	Sampled Terminal (n)	Survival, n/6 (%)
1	0.1	1	1.36 × 10^10^	4.16 ± 0.31	4	2	4/6 (67)
2	0.1	2	1.36 × 10^10^	3.84 ± 0.36	4	2	4/6 (67)
3	0.1	3	1.36 × 10^10^	3.53 ± 0.42	2	4	2/6 (33)
4	0.2	1	2.72 × 10^10^	3.90 ± 0.39	2	4	2/6 (33)
5	0.2	2	2.72 × 10^10^	3.58 ± 0.34	6	0	6/6 (100)
6	0.2	3	2.72 × 10^10^	3.21 ± 0.30	6	0	6/6 (100)
7	0.3	1	4.08 × 10^10^	3.65 ± 0.37	5	1	5/6 (83)
8	0.3	2	4.08 × 10^10^	3.37 ± 0.32	6	0	6/6 (100)
9	0.3	3	4.08 × 10^10^	3.02 ± 0.29	6	0	6/6 (100)
10	0.5	1	6.80 × 10^10^	3.40 ± 0.33	6	0	6/6 (100)
11	0.5	2	6.80 × 10^10^	3.09 ± 0.28	6	0	6/6 (100)
**12**	**0.5**	**3**	**6.80 × 10^10^**	**2.64 ± 0.25**	**6**	**0**	**6/6 (100)**
Infected control	**—**	**—**	**—**	4.41 ± 0.45	1	5	1/6 (17)

^1^ n = 6 animals per group. Sampled day 5 (n), spleens collected from animals surviving to day 5; sampled terminal (n), spleens collected at death or at a humane endpoint. Body-weight data are reported in Section 3.7 and are deliberately not pooled across these two sampling intervals. Bold indicates the best-performing regimen, which formed the primary comparison with the infected untreated control group. —, not applicable.

## Data Availability

The 16S rRNA gene sequence of *Klebsiella pneumoniae* KP-MB-KAZ34 has been deposited in GenBank under accession number PZ656399. All other data supporting the findings of this study are contained within the article and the Supplementary Materials. Further inquiries can be directed to the corresponding author.

## Supplementary Materials

The following supporting information can be downloaded at: https://www.mdpi.com/article/10.3390/biotech15040084/s1, Table S1: Antimicrobial susceptibility profile of *Klebsiella pneumoniae* KP-MB-KAZ34 determined by the disk diffusion method.

## Abbreviations

The following abbreviations are used in this manuscript:

CRKP	Carbapenem-resistant *Klebsiella pneumoniae*
CFU	Colony-forming units
EU	Endotoxin units
EUCAST	European Committee on Antimicrobial Susceptibility Testing
GRM	Dry nutrient medium based on fish-meal hydrolysate
ICTV	International Committee on Taxonomy of Viruses
MDR	Multidrug-resistant
MOI	Multiplicity of infection
PFU	Plaque-forming units
16S rRNA	16S ribosomal RNA
