# Supplementary material for: Phenotypic Characterization and Preliminary In Vivo Evaluation of a Locally Isolated Klebsiella pneumoniae Bacteriophage
Source: BioTech (Basel). 2026 Sep 29;15(4):84. doi: 10.3390/biotech15040084 (PMC13702762; doi:10.3390/biotech15040084)
Supplement: Supplementary file 1 [file biotech-15-00084-s001.zip › biotech-4489805-supplementary.pdf]

*Article*

# Phenotypic Characterization and Preliminary In Vivo Evaluation of a Locally Isolated *Klebsiella pneumoniae* Bacteriophage

**Table S1.** Antimicrobial susceptibility profile of *Klebsiella pneumoniae* KP-MB-KAZ34 determined by the disk diffusion method.

| Antimicrobial class | Antimicrobial agent | Disc content, μg | Inhibition zone, mm | EUCAST interpretation |
|---|---|---|---|---|
| Aminopenicillins | Ampicillin | 10 | 6 | R |
| Penicillin/β-lactamase inhibitor combination | Amoxicillin-clavulanic acid | 20/10 | 14 | R |
| Third-generation cephalosporins | Cefotaxime | 5 | 12 | R |
| Third-generation cephalosporins | Ceftazidime | 10 | 15 | R |
| Fourth-generation cephalosporins | Cefepime | 30 | 20 | R |
| Carbapenems | Imipenem | 10 | 17 | R |
| Carbapenems | Meropenem | 10 | 14 | R |
| Fluoroquinolones | Ciprofloxacin | 5 | 16 | R |
| Fluoroquinolones | Levofloxacin | 5 | 17 | R |
| Aminoglycosides | Gentamicin | 10 | 14 | R |
| Aminoglycosides | Amikacin | 30 | 21 | S |

Antimicrobial susceptibility testing was performed by the disk diffusion method on Mueller-Hinton agar using an inoculum adjusted to 0.5 McFarland standard. Plates were incubated aerobically at 35 ± 1°C for 18 ± 2 h. Inhibition-zone diameters were interpreted according to the EUCAST Clinical Breakpoint Tables, version 15.0, for Enterobacterales. *Escherichia coli* ATCC 25922 was used as the quality-control strain. S, susceptible at standard exposure; R, resistant.
